# Biocompatible Hydrogels for Microarray Cell Printing and Encapsulation

**DOI:** 10.3390/bios5040647

**Published:** 2015-10-26

**Authors:** Akshata Datar, Pranav Joshi, Moo-Yeal Lee

**Affiliations:** Department of Chemical & Biomedical Engineering, Cleveland State University, 1960 East 24th Street Cleveland, OH 44115-2214, USA; E-Mails: a.datar17@vikes.csuohio.edu (A.D.); p.joshi18@vikes.csuohio.edu (P.J.)

**Keywords:** microarray, cell encapsulation, hydrogel, bioprinting, miniaturized 3D cell culture

## Abstract

Conventional drug screening processes are a time-consuming and expensive endeavor, but highly rewarding when they are successful. To identify promising lead compounds, millions of compounds are traditionally screened against therapeutic targets on human cells grown on the surface of 96-wells. These two-dimensional (2D) cell monolayers are physiologically irrelevant, thus, often providing false-positive or false-negative results, when compared to cells grown in three-dimensional (3D) structures such as hydrogel droplets. However, 3D cell culture systems are not easily amenable to high-throughput screening (HTS), thus inherently low throughput, and requiring relatively large volume for cell-based assays. In addition, it is difficult to control cellular microenvironments and hard to obtain reliable cell images due to focus position and transparency issues. To overcome these problems, miniaturized 3D cell cultures in hydrogels were developed via cell printing techniques where cell spots in hydrogels can be arrayed on the surface of glass slides or plastic chips by microarray spotters and cultured in growth media to form cells encapsulated 3D droplets for various cell-based assays. These approaches can dramatically reduce assay volume, provide accurate control over cellular microenvironments, and allow us to obtain clear 3D cell images for high-content imaging (HCI). In this review, several hydrogels that are compatible to microarray printing robots are discussed for miniaturized 3D cell cultures.

## 1. Introduction

In the process of drug discovery, great importance is given to high-throughput, *in vitro* cell-based assays with the capability of high-content imaging (HCI) [1]. Conventional cell monolayers cultured on the surface of 96-wells (also known as, 2D cell monolayer cultures) have been widely used as a gold standard for cellular *in vitro* models in high-throughput screening (HTS) of compounds. However, 2D cell monolayer cultures may not accurately mimic physiological properties of tissues *in vivo*, thus providing limited predictability of drug responses [2]. To address this issue and better mimic cellular microenvironments in tissues, three-dimensional (3D) cell cultures have been adopted in preclinical evaluations of drug candidates. These have several advantages over the conventional 2D cultures, including *in-vivo*-like cell morphology with cell-cell and cell-extracellular matrix (ECM) interactions and physiologically-relevant gene/protein expression [3,4]. Although there have been several methods developed to grow cells in 3D, including microbeads, 3D scaffolds, cells encapsulated in hydrogels, and hanging droplets [4,5,6,7], in this review we focus on miniaturized 3D cell cultures in hydrogels that are compatible with microarray bioprinting.

Microarray bioprinting refers to printing cells encapsulated in hydrogels in a spatially addressable manner using automated liquid dispensing robots such as microarray spotters. Cells in nano-liter volume (as small as 30–60 nL) can be dispensed onto functionalized glass slides or micropillar/microwell chip platforms and grown in growth media to support miniaturized 3D cell cultures for toxicology assays [8,9]. For example, cells in hydrogels can be spotted or printed on top of the micropillars and immersed into the microwells containing growth media for miniaturized 3D cell cultures. The microwell chip can accommodate up to 950 nL of compounds, recombinant viruses, growth factors, and fluorescent dyes for various cell-based assays. Since the micropillar chip is complementary to the microwell chip, cells on the micropillars can be exposed to hundreds of different test conditions in the microwells simultaneously by simply sandwiching the two chips together [9]. The cells on the micropillar chip can be exposed to compounds for a period of time and stained with fluorescent dyes or fluorescently labeled antibodies to assess drug efficacy and toxicity [9]. Compared to traditional 2D cell monolayer cultures, microarray bioprinting offers several attractive features, including physiologically relevant cells grown in 3D, miniaturization of cell-based assays saving valuable raw materials, such as primary human cells obtained from patients, and ultrahigh-throughput capability of testing cell culture conditions [10].

In this review, we address the fundamental principles of microarray bioprinting, the roles of hydrogels for cell encapsulation, the properties of hydrogels required for microarray bioprinting, and a few examples of hydrogels that can meet the requirements.

## 2. Microarray Bioprinting Technology

**Figure 1:**
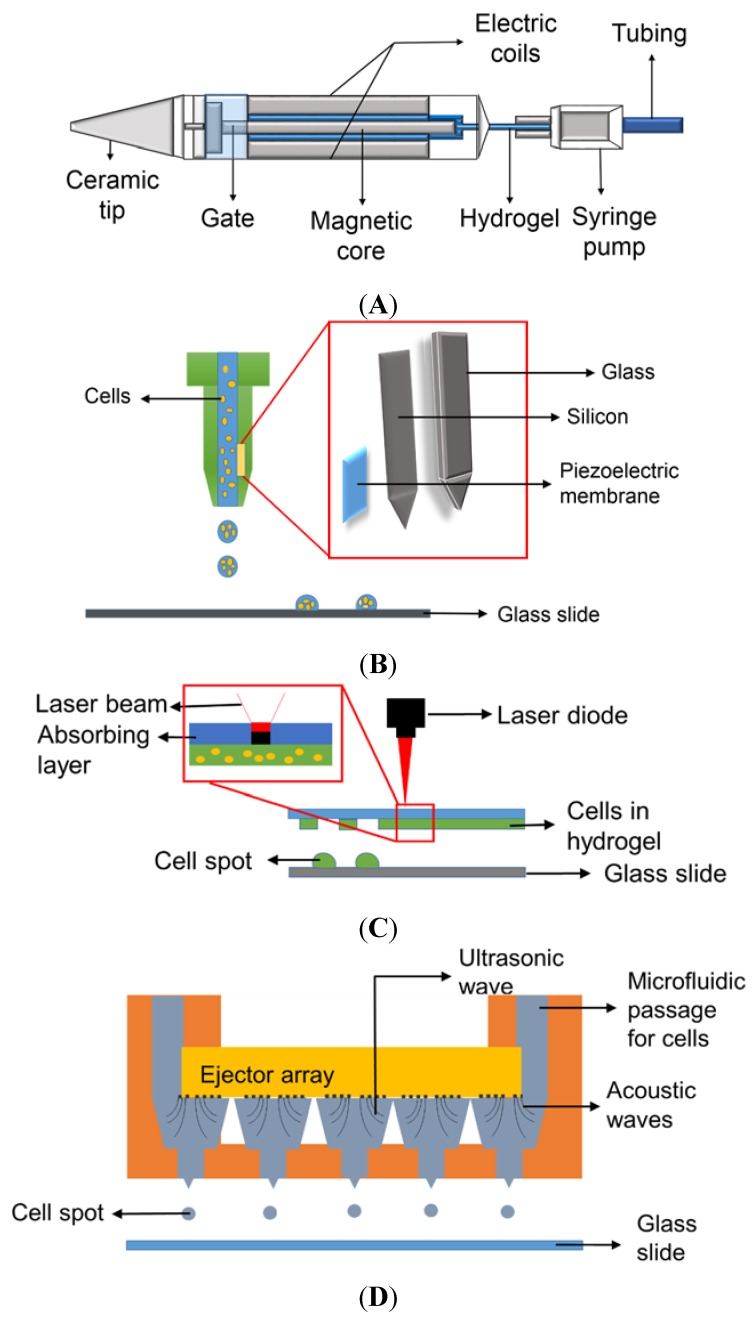
Various mechanisms for printing biological samples: (**A**) Micro-solenoid valve using electromagnetic induction; (**B**) Piezoelectric nozzle using piezoelectric vibration; (**C**) Laser-induced forward transfer (LIFT) using a laser beam to propel cell spots [13], and (**D**) Acoustic wave generator using ultrasound to produce acoustic waves for cell printing (Reproduced from Reference [23] with permission of The Royal Society of Chemistry).

To encapsulate cells in 3D and prevent their direct contact with the surface, polymeric substances (hydrogels) that have capacity to hold a large amount of water and show compatibility to cells have been employed in microarray bioprinting. Hydrogels can contain growth media and growth factors to support cell growth for 3D cell cultures and various other applications [11,12]. These cells in hydrogels can be dispensed on glass slides or plastic chips via several printing technologies, including micro-solenoid valves, piezoelectric nozzles, and laser-induced forward transfer (LIFT) technique, and acoustic bioprinting [13]. Among these, micro-solenoid valves (Figure 1A) working on the principle of electromagnetic induction are the most commonly used for cell printing due to their robust and reliable printing with cells in hydrogels [8]. A micro-solenoid valve consists of a metal rod within solenoid coils that moves up and down by electric voltages applied, acting as a gate to dispense cells in hydrogels. The intensity and duration of voltage applied to the micro-solenoid valve control the open time of the gate and hence determine the volume of biological samples dispensed. Certain pressure is applied to maintain the liquid sample to move forward when the gate is opened; typically syringe pumps are necessary to maintain the pressure and dispense cells in hydrogels. The solenoid valves allow us to print relatively high density of cells in hydrogels and accommodate relatively viscous samples. However, the dispensing volume is large, ranging from 20 to 1000 nL, compared to piezoelectric nozzles. The principle behind piezoelectric nozzles (Figure 1B) is very similar to the conventional inkjet printers [14]. The piezoelectric transducer in the nozzle contracts and expands with application of certain voltages, which pushes biological samples including cells in hydrogels to flow [15]. The volume dispensed depends on the voltage and frequency applied, viscosity of hydrogels, and the diameter of the nozzle, making the dispensing volume extremely small, ranging from 50 to 1000 pL. However, piezoelectric printing is significantly influenced by viscosity of the samples, and the nozzles are frequently clogged with cells. Thermal inkjet printers can be used to print cells in hydrogels in a high throughput fashion and may reduce the tip clogging issue considerably by heating polymers and making them less viscous. However, thermal inkjet printing may not be suitable for cell printing due to cell damage or death by high temperature [16]. Another printing technology that can be used for microarray bioprinting is laser-induced forward transfer (LIFT) (Figure 1C). In this technique, a donor film and an acceptor film are set in a parallel manner, where the donor film is a thin film made of cells in hydrogel to be printed. A laser beam is shone on the absorbing substrate layer, which develops laser-induced vapor bubbles, inducing deposition of cell spots on the acceptor film that is either cell culture media or biopolymer-coated glass slides [13,15,17]. Interestingly, the volume of the cell droplets dispensed using this technique is varied, depending on the temperature applied, the nature of biological samples, and the thickness of the donor film containing cells [15,18]. With appropriate optimization of laser beam intensity and focusing conditions, the LIFT technology has been applied for printing DNA, proteins, peptides, and cells in microarrays [14,19]. Although LIFT can be used to print a wide range of biological samples, cell printing in hydrogels with LIFT might be challenging due to high temperature induced by the laser beam. Therefore, maintaining high cell viability and proliferation after printing would be a concern [20,21]. As the dispensed volume depends on the thickness of the hydrogel layer, the uniformity of cell seeding and cell distribution over the glass slide have to be investigated and validated prior to data analysis with test compounds. Acoustic printers depend on ultrasound for printing cells in hydrogels (Figure 1D). A high-intensity acoustic wave is generated by focusing ultrasound beams, and this energy is used to dispense liquid droplets from air-liquid interface. This technique was initially developed for printing single cells in pico-liter droplets. However, modification and optimization have enabled the implementation of 3D cell cultures [22,23]. Characteristics of bioprinting methods are summarized in Table 1. Cell printing using acoustic printers may be questionable as cell membrane might be affected and ruptured when the cells are exposed to ultrasonic waves and, hence, this technique is considered unsuitable for this application [24].

**Table 1:** Comparison of typical microarray bioprinting methods.

Printing Mechanisms	Cell Viability	Hydrogels Printed	Cells Printed	Spot Volume (nL)	References
Micro-Solenoid Valve	>95%	Alginate, polyvinyl alcohol, PuraMatrix^™^, Matrigel^®^	Hela cell line, human glioblastoma, hepatoma cell lines	20–1000	[8,9,14,25,26]
Piezoelectric Nozzle	95%	Polyethylene glycol diacrylate (PEG-DA), methacrylated gelatin	Human fibroblasts, chondrocytes, HepG2	0.05–1	[13,14,27,28]
LIFT	>90%	Gelatin, Matrigel^®^	Human mesenchymal stem cells, keratinocytes	0.1	[13,27,29]
Ultrasonic Wave Generator	85%–89%	Dextran, polyethylene glycol (PEG)	Mouse myofibroblasts, embryonic stem cells, breast cancer cell lines, cardiomyocytes	40–300	[12,22]

Several micro-solenoid valves or piezoelectric nozzles are mounted on a robotic arm that moves along X, Y, and Z axis and works simultaneously to print biological samples, which is called a microarray spotter [30]. There are several clear advantages of using the microarray spotter, including its accurate printing of very small volumes of biological samples such as cells in hydrogels, growth media, growth factors, recombinant viruses, proteins, and DNAs. In addition, it can reduce manual intervention by controlling cell dispensing with a computer in high throughput. Preventing evaporation of water in extremely small droplets can be done with surface chilling and condensation, and high speed printing [5,9,14]. However, there are several drawbacks in the microarray spotters. The printing tips can be easily clogged when big particles (e.g., microbial strains) are printed or highly viscous hydrogels are used to encapsulate cells [9]. To minimize the clogging issues, cells have to be well suspended in hydrogels immediately before printing, and a proper seeding density of cells has to be tested and used. Furthermore, rapid gelation of hydrogels within micro-solenoid valves, piezoelectric nozzles, ceramic printing tips, and tubing have to be avoided. Thus, it is extremely critical to understand the mechanisms of hydrogel gelation and optimum conditions used prior to cell printing using the microarray spotter [9,31,32]. The criteria of hydrogel selection are primarily based on the mechanism of gelation, compatibility with cells, and microarray spotters used. Several applications of the microarray bioprinting technology have been demonstrated, which include high-throughput assessment of metabolism-induced toxicity [8], screening of anticancer drugs [9], stem cell differentiation [33], and miniaturized tissue engineering by layer-by-layer cell printing [34].

## 3. Factors to be Considered When Selecting Hydrogels for Microarray Bioprinting

When selecting hydrogels for microarray bioprinting, several critical factors such as gelation mechanism, compatibility with surface, and biocompatibility have to be considered. The mechanism of gelation is one of the most important aspects of hydrogels for successful microarray bioprinting [35]. Without complete understanding of the gelation mechanism, it is difficult to assess feasibility of hydrogels for printing. In general, temperature-sensitive hydrogels, such as Matrigel^®^, require micro-solenoid valves and tubing to be chilled to avoid spontaneous gelation within the system at room temperature. Once gelation takes place in micro-solenoid valves, it is almost impossible to remove the hydrogel from the valves. Thus, gelation of hydrogel by ionic crosslinking [36], photo-polymerization [37], biocatalysis [38], covalent bonding [39], or pH-induced phase transition [38] would be better suited for cell encapsulation in microarray bioprinting [40]. Typically, gelation of hydrogels with cells on a glass slide or a plastic chip takes place by printing two components necessary for gelation sequentially. For example, barium chloride or calcium chloride that is commonly used to crosslink alginate is printed on top of micropillars, which is followed by printing alginate mixed with cells on the surface of the salt to initiate gelation [8,41]. This approach is simple and ideally suited to minimize unwanted gelation in micro-solenoid valves and remove remaining hydrogels by rinsing with water. One of the drawbacks of these types of ionic hydrogels is that they degrade slowly over time by chelating ions such as phosphate ions in growth media. UV irradiation on photo initiators combined with hydrogels can cause phase transition and be used to encapsulate cells [42,43]. However, long-term irradiation of UV can affect the viability of cells encapsulated on the glass slide [43,44]. Covalent crosslinking by radical reactions can be employed for microarray bioprinting as well. However, these chemicals can be toxic to the cells [31,42]. In addition, surface charge of hydrogels is an important factor to avoid ionic interactions between compounds and hydrogels used. Negatively or positively charged compounds can interact with certain functional groups (e.g., –COOH group to –NH_2_ group) on hydrogels and even adsorbed to hydrogels or the surface of a functionalized glass slide. There has been no universal hydrogel developed yet that can be used for all types of cells because each hydrogel has different cyto-compatibility and mechanisms of gelation.

**Figure 2:**
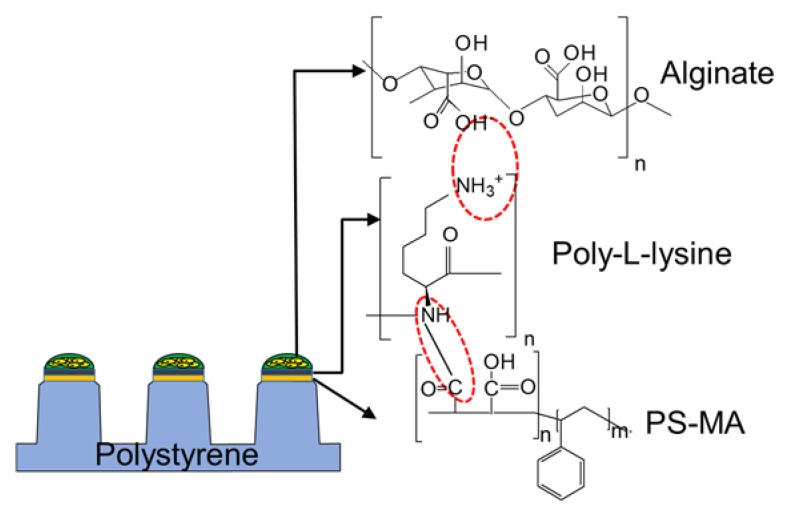
Chemical and ionic interactions among polystyrene-*co*-maleic anhydride (PS-MA), poly-l-lysine (PLL), and alginate that support cell spots to adhere to the surface of the micropillar chip.

In addition to the mechanism of gelation and biocompatibility, the interaction between the hydrogel used for cell encapsulation and the surface where the cell spots are attached becomes critical, to avoid detachment of the cell spots from the surface. For example, cell spots can be encapsulated in alginate and attached on the surface of the micropillar chip through polystyrene-*co*-maleic anhydride (PS-MA), poly-L-lysine (PLL), and alginate interactions (Figure 2) [8]. The micropillar chip made of polystyrene is hydrophobic, which makes the surface incompatible with hydrophilic hydrogel spots. Thus, the micropillar chip requires coating with an amphiphilic functional polymer, such as PS-MA, that is strongly attached on the surface of polystyrene through hydrophobic interactions. PLL is dispensed on the PS-MA coating to enhance cell spot attachment. Due to reactivity of maleic anhydride to amine groups on PLL, PLL printed on the tip of the micropillars can be covalently attached. Finally, PLL can interact with negatively charged alginate spots with cells due to positive charges remaining on PLL [8]. Alginate with cells can form a gel with barium chloride printed with PLL on the chip. Additionally, physicochemical interactions between printed biomaterials and the chip surface is another factor to consider. For example, thrombin that is printed on the micropillar chip to initiate gelation of fibrin gel can be denatured on the hydrophobic nature of the chip surface [45]. Thus, changing the surface property to hydrophilic by coating or printing hydrophilic biopolymers would be critical to minimize denaturation of thrombin.

The ability to manipulate cellular microenvironments becomes critically important in miniaturized 3D cell cultures, which include the specific arrangement of cell types, composition of extracellular matrices (ECMs), gradients of soluble factors (growth and differentiation factors), and cell encapsulation materials (natural hydrogels and synthetic polymers) [43,46]. In particular, additives in hydrogels such as ECMs, including elastin, collagen, fibronectin, proteoglycans, laminin and hyaluronic acid can influence cell viability, proliferation and differentiation by controlling signaling pathways in the cells [47]. The ECM-cell interactions are required to better mimic 3D microenvironments [35]. Thus, hydrogels selected for cell encapsulation have to be compatible with ECMs as well. Some highly charged hydrogels tend to be precipitated when mixed with ECMs, which are unsuited for microarray bioprinting.

Mechanical stability over time as well as transparency of hydrogels have to be considered for microarray bioprinting to obtain proper cell images from staining with fluorescent dyes or fluorescently-labeled antibodies [48,49]. Mammalian cells encapsulated in hydrogels tend to secrete matrix metalloproteinases (MMPs) over time to get accustomed to a new microenvironment [50,51]. These MMPs can degrade protein/peptide-based hydrogels and weaken mechanical stability of hydrogels resulting in cell spot detachment during incubation or cell staining. Therefore, degradable hydrogels such as Matrigel^®^ and PuraMatrix^™^ have to be mixed with non-degradable hydrogels, such as alginate, when cells are cultured long-term. In addition, transparency of hydrogels is important for high-content cell imaging to minimize auto-fluorescence. Unlike applications of hydrogels in tissue engineering [45], swelling of hydrogels is not a concern due to small volume of cell spots printed.

## 4. Potential Hydrogels for Microarray Cell Printing and Encapsulation

Based on their origin, hydrogels are classified into natural and synthetic polymers [52,53]. Natural hydrogels include alginate, collagen, Matrigel^®^, fibrin, agarose, and gelatin among others. Synthetic hydrogels include polyethylene glycol (PEG), polyvinyl alcohol (PVA), polyacrylate, and polyurethane [54], which are usually preferred when increased mechanical strength is required [52,55,56]. A few examples of hydrogels that have been used for microarray cell printing and those we think can be potentially used for cell encapsulation on the chip are discussed.

### 4.1. Natural Hydrogels

#### 4.1.1. Alginate

Alginate is a natural polymer derived from brown sea weed, and has been widely used in tissue engineering applications due to its biocompatibility, availability, and low cost [41]. Negatively charged alginate forms a transparent gel with divalent cations such as Ba^2+^, Ca^2+^, and Mg^2+^ via ionic crosslinking [8]. Its applications include encapsulation of human embryonic stem cells (hESCs), bone tissue regeneration, development of liver cell models, and drug efficacy/toxicity testing [57,58,59]. Cells encapsulated in alginate have shown good viability due to diffusion of nutrients and gases through the alginate gel and nontoxic nature of the alginate matrix to cells [60]. Alginate stands out as a good hydrogel as it supports cell growth and proliferation and is potentially non-toxic. Although alginate is easy to print at low concentrations (e.g., 1%) and non-degradable by MMPs, there are several limitations that might prevent its wide range of use. For example, high concentrations of divalent cations used for its gelation can be toxic to cells, thus necessitating the cells rinsed with growth media after encapsulation. Alginate is susceptible to degradation by chelating agents, leading to mechanical instability over time. In addition, alginate does not support viral transduction of cells due to high affinity of recombinant viruses to the alginate matrix [26,31,41]. Efforts have been made to make alginate non-degradable by chemical modification with methacrylate, which make alginate photo-crosslinkable while maintaining low cytotoxicity, and hence a reliable hydrogel for cell encapsulation [42]. Although alginate is widely used for cell encapsulation *in vitro*, there is no interaction between cells and alginate matrix, causing retarded cell growth or death [61]. Alginate may also cause inflammatory responses in the presence of immune cells, which can limit the applications of alginate-based cell encapsulation *in vivo* [62].

#### 4.1.2. Matrigel^®^

Matrigel^®^ is a mixture of basement membrane proteins extracted from Engelbreth-Holm Swarm (EHS) mouse sarcoma cells, consisting of laminin, collagen IV, entactin, and heparin sulfate proteoglycan along with various growth factors [63]. Since it resembles complex cellular microenvironments found in many tissues, it has been widely used in cell growth, differentiation, angiogenesis, and tissue vascularization [64,65]. Unlike alginate, Matrigel^®^ is a temperature sensitive hydrogel which forms a transparent gel at a temperature ranging from 24 to 37 °C, with the speed of gelation being dependent on concentration and incubation temperature [66]. The mechanical properties of Matrigel^®^ can be enhanced by glutaraldehyde crosslinking [45]. For microarray printing, cells are mixed with cold Matrigel^®^ on ice and then printed immediately while maintaining the dispensing head and tubing below 9 °C. Printed cell spots on the chip is gelled at 37 °C in a humid incubation chamber. Printing Matrigel^®^ requires repeated rinsing of tubing with cold water to maintain low temperature and prevent undesirable gelation in the tubing, which is cumbersome and difficult. Another limitation of Matrigel^®^ comes from batch-to-batch variations in its compositions due to differences in the size of tumor and tissue preparation, which greatly affect reliability and reproducibility of experimental outcomes. Due to this variation, some batches of Matrigel^®^ tend to form a gel quicker than the others [63,66]. Finally, unidentified growth factors included in Matrigel^®^ can influence cell differentiation, which limits the use of Matrigel^®^ for stem cell research.

#### 4.1.3. Fibrin

Fibrinogen is a large and complex glycoprotein that is converted into fibrin due to thrombin driven polymerization during blood clot formation [52]. Fibrin gel has been widely used in gene delivery, cell growth and differentiation, and tissue engineering to fill bone cavities and repair neurons, heart valves, vascular grafts, and the surface of the eye [47,57,67,68]. The rate of gelation is strongly influenced by the concentration of fibrinogen and the activity of thrombin. For microarray bioprinting, thrombin is printed on the surface of the micropillar chip first, and then a mixture of cells and fibrinogen is printed on top of thrombin spots. Thrombin initiates polymerization of fibrinogen on the chip, forming a transparent gel with cells. However, the transparency of the gel depends on the concentration of fibrinogen and thrombin used [67]. Similar to Matrigel^®^, fibrin gel can be degraded by proteolytic enzymes such as MMPs, which may lead to instability of gel structures over time. To minimize or control degradation of fibrin gel, proteinase inhibitors, such as aprotinin, are added in growth media [67,69]. In addition, the mechanical strength of fibrin gel can be enhanced by supplementing Ca^2+^ ions [57].

#### 4.1.4. Collagen

Collagen is the main structural protein found in various connective tissues and the most abundant protein in mammals [70]. Among various types of collagen found, type I collagen is the most commonly used for 3D cell cultures in tissue engineering [41,65,71]. In addition, type IV collagen found in Matrigel^®^ provides structural support to the matrix and assembles other basement membrane components through interactions. Collagen spontaneously forms a triple helix scaffold at neutral pH and 37 °C, leading to gelation [72]. Although collagen is one of the most well-known and biocompatible hydrogels, it is easily broken down by collagenases and other proteolytic enzymes secreted by cells [4,71]. Since collagen is a temperature sensitive hydrogel, the protocol for microarray printing is similar to that of Matrigel^®^ and share the same limitations [3,63,73].

#### 4.1.5. Hyaluronic Acid

Hyaluronic acid (HA) is an anionic, non-sulfated glycosaminoglycan commonly found in connective, epithelial, and neural tissues. Because of its common presence in ECMs and influence in signaling pathways through interactions with cell surface receptors, hyaluronic acid has been widely used as a scaffold material in tissue engineering and regenerative medicine, in particular for stem cell differentiation, wound healing, and angiogenesis [35]. However, hyaluronic acid typically shows poor mechanical strength and can be broken down by hyaluronidases, thus requires crosslinking with chemical functional groups [35]. For example, thiol groups for Extracel and HyStem, hexadecylamide for Hymovis, tyramine for Corgel, formaldehyde for Hylan-A, and divinylsulfone for Hylan-B have been attached on hyaluronic acid for gelation [74,75]. Extracel is a mixture of Gelin-S, Glycosil, and Extralink in 2:2:1 ratio [43], among which Glycosil is thiol-modified hyaluronic acid and Gelin-S is thiol-modified gelatin. Extralink is a chemical cross linker, which can influence viscosity of Extracel through crosslinking. The mixture of Glycosil and Extralink alone can form a gel, but Gelin-S is added to enhance the viability of the cells encapsulated in Extracel [43,76]. Corgel is another hyaluronic acid-derived hydrogel, and gelation occurs due to crosslinking of tyramine-functionalized HA in the presence of peroxidases [75]. Corgel has been used for tissue regeneration in rat models [77]. To further enhance its biocompatibility, hyaluronic acid has been combined with fibronectin for culturing endothelial cells in wound healing and angiogenesis [37]. These hybrid hydrogels are used to encapsulate chondrocytes, adipocyte stem cells, and mesenchymal stem cells (MSCs) as well [43,78]. High viscosity of hyaluronic acid may cause clogging of tips or solenoid valves in microarray spotters. Similar to alginate, it is not very cell adhesive, thus requiring further chemical modification to provide cell-matrix interactions [52].

### 4.2. Synthetic Hydrogels

#### 4.2.1. PuraMatrix^™^

PuraMatrix^™^ is a biocompatible, synthetic peptide, which is self-assembled when mixed with small amounts of salts in cell culture media and form a transparent gel [79]. It mimics natural ECMs and allows cell proliferation in 3D cultures. In addition to this it is known for neurite outgrowth, active synaptic formation and maintaining embryonic stem cells undifferentiated [79]. It is stable at wide ranges of temperature and pH and supports the growth of various cells in tissue engineering scaffolds [36]. For microarray bioprinting, cells have to be rinsed with 10% sucrose and then pelleted by centrifugation to remove salts in cell suspension. Various salt solutions can be printed first on the chip to initiate gelation, which is followed by printing a mixture of PuraMatrix^™^ and sucrose-rinsed cell pellets. Cell pellets have to be handled gently to avoid cell membrane rupture when mixing with PuraMatrix^™^. One of critical limitations of PuraMatrix^™^ is its low pH [79], which is harmful to many cells, leading to low cell viability after encapsulation. Thus, the mixture of PuraMatrix^™^ and cells has to be printed quickly and rinsed with growth media to neutralize pH [76]. This cumbersome handling and medium changing increase the risk of cell contamination.

#### 4.2.2. Polyethylene Glycol (PEG)

PEG is one of the most widely studied synthetic polymers in cell encapsulation due to its high water solubility, non-immunogenic response, low toxicity, and non-biodegradable property [54]. Various derivatives of PEG have been used to encapsulate cells for biomedical applications [55]. Gelation of PEG derivatives is initiated by either UV irradiation or simple redox reactions [80]. These polymers provide desirable mechanical strength and other properties for tissue engineering, but often lack biocompatibility necessary to support cell proliferation and differentiation [81]. To overcome this limitation, various efforts have been made to develop biocompatible derivatives with nontoxic natural polymers [66]. PEG-based scaffolds are capable of cell encapsulation and result in better predictive responses in testing efficacy and toxicity of breast cancer drugs, compared to conventional 2D cell monolayer approaches [82]. The crosslinking mechanisms of the hydrogels have been compared in Table 2.

**Table 2:** Summary of hydrogels compatible for high-throughput cell printing and encapsulation.

Hydrogel	Gelation Mechanism	Compatible Cell Lines	Advantages	Limitations
Alginate	Crosslinking via divalent ions	Human adipose derived stem cells [61], human brain cancer cells [9]	Good printability, applicable to stem cell growth [61], easy chemical modification [52]	Non-supportive to viral transduction [26]
Matrigel^®^	Temperature dependent	Human umbilical vein endothelial cells, colorectal cancer cells [63], rat cardiomyocytes [64]	Applicable to differentiation, xenografts, spheroidal cell growth, 3D co-cultures [63]	Clogging tips and solenoid valves due to temperature sensitive gelation
Fibrin	Thrombin catalyzed polymerization	Chondrocytes [57], rat myoblast [68]	Applicable to tissue engineering, vascular grafts, gene delivery [35,47]	Unstable due to degradation via MMPs [67]
Collagen (type 1)	Temperature induced	Endothelial progenitor cells, mesenchymal progenitor cells [69]	Applicable to tissue engineering [73]	Unstable due to degradation via collagenases, clogging tips and valves [83]
Hyaluronin	Thermal or photo dependent gelation [52]	Mesenchymal stem cell [35], endothelial cells [37]	Applicable to tissue engineering, regenerative medicine, stem cell differentiation, wound healing, angiogenesis [76]	Poor mechanical strength
PuraMatrix^™^	Self-assembling when exposed to salts	Primary rat hepatocytes, adult liver progenitor cells, chondrocytes [79]	Embryonic stem cell cultures [79]	Poor cyto-compatibility due to low pH
PEG	UV crosslinking, simple redox crosslinking	Human mesenchymal stem cells [54]	High mechanical strength	No cell-matrix interaction

## 5. Summary

Microarray bioprinting is a promising high-throughput approach for miniaturized 3D cell cultures. The reduction in sample volume and the speed of printing hundreds of samples in a short period of time make this technology suitable for wide ranges of cell-based assays. The major concern however, is to find out appropriate hydrogel matrices for cell encapsulation which provide tunability of their physical and chemical properties while being relatively cost-effective. Natural hydrogels are preferred due to high biocompatibility, mild gelation, and flexibility for chemical modification. However, several limitations such as poor control over material properties and mechanical strength make these hydrogels inconvenient when high mechanical properties are desired. Synthetic hydrogels provide better control over physical and chemical properties, but their use can be limited due to lack of biocompatibility. The selection of an optimum hydrogel matrix depends on specific applications. With wide options for selecting suitable hydrogels for miniaturized 3D cell cultures, researchers can optimize gelation and cell culture conditions for microarray bioprinting.
